# Treatment of newly diagnosed moderate or severe chronic graft-versus-host disease with prednisone and everolimus (PredEver first): a prospective multicenter phase IIA study

**DOI:** 10.1038/s41409-024-02289-0

**Published:** 2024-05-02

**Authors:** Francis Ayuk, Eva-Maria Wagner-Drouet, Daniel Wolff, Natascha von Huenerbein, Ute-Marie von Pein, Evgeny Klyuchnikov, Stephanie von Harsdorf, Christian Koenecke, Herbert Sayer, Nicolaus Kröger

**Affiliations:** 1 Department of Stem Cell Transplantation, University Medical Center Hamburg-Eppendorf, Hamburg, Germany; 2 Department of Hematology, Medical Oncology, and Pneumology, University Medical Center, Mainz, Germany; 3 Department of Internal Medicine III, Hematology and Oncology, University Medical Center Regensburg, Regensburg, Germany; 4 Department of Internal Medicine III, University Hospital of Ulm, Ulm, Germany; 5 Department of Hematology, Hemostasis, Oncology and Stem Cell Transplantation, MHH, Hanover, Germany; 6 Department of Internal Medicine II, University Medical Center Jena, Jena, Germany; 7 Present Address: Department of Medicine, Diakonie Hospital, Stuttgart, Germany; 8 Present Address: Department of Hematology, Oncology and Stem Cell Transplantation, Helios Klinikum Erfurt, University Campus of the Health and Medical University, Potsdam, Germany

**Keywords:** Diseases, Signs and symptoms

## Abstract

Although most patients with chronic graft-versus-host disease (cGVHD) show initial response to first-line therapy, long-term clinically meaningful success of first-line treatment remains rare. In a prospective multicentre phase II trial in 6 German centers, patients with newly diagnosed moderate or severe cGVHD received prednisone and everolimus for 12 months followed by a 1-year follow-up period. Primary endpoint was treatment success (TS) at 6 months defined as patient being alive, achieving PR or CR of cGVHD, having no relapse of underlying disease and requiring no secondary treatment for cGVHD. Of the 34 patients evaluable for efficacy, 19 (56%) had TS at 6 months with 22 and 52% of the patients in a CR and PR respectively. Overall 30 patients (88%) had a CR or PR as best response, nearly all responses (29/30) occurring within the first 6 weeks of treatment. The cumulative incidence of treatment failure at 1 year was 63%, corresponding to 37% TS. Predefined safety endpoint (thrombotic microangiopathy, pneumonitis, and avascular necrosis) were not observed in any patient. Addition of everolimus to prednisolone is well tolerated and may improve long-term treatment success. Larger studies are necessary to ascertain the possible role of everolimus in first-line treatment of cGVHD.

## Introduction

Chronic graft-versus-host disease (cGVHD) is a common long-term complication of allogeneic hematopoietic stem cell transplantation (allo-HSCT) and associated with impaired immunity, compromised functional status, and quality of life making it a leading cause of morbidity and mortality beyond day 100 after allo-HSCT [1–3].

Because of the deleterious side effects of protracted systemic steroid treatment, efforts have been made to combine steroids with other drugs in order to spare steroids and possibly improve efficacy while reducing side effects. However, the results of these studies in treatment of newly diagnosed cGVHD have so far been disappointing with no improvement in efficacy or survival [4–7].

The expanding therapeutic arsenal of cGVHD now includes many other agents that have been evaluated mostly in second-line therapy and beyond such as mTOR inhibitors, Jak inhibitors, ROCK2 inhibitors, BTK inhibitors, proteasome inhibitors monoclonal antibodies, and extracorporeal photopheresis amongst others [8, 9]. The median duration of immunosuppression of 23 months and the high 3-year non-relapse mortality of up to 40% [10] emphasize the urgent need for new first-line treatment strategies for patients with cGVHD.

Several small phase II trials studying mTOR inhibitors (everolimus and sirolimus) in the treatment of refractory cGVHD have reported response rates of up to 81% in combination with CNI, corticosteroids or MMF [11–16]. Major side effects such as thrombotic microangiopathy (TMA) and nephropathy were mainly observed in combination with CNI. Experience from solid organ transplant, cGVHD studies, and data from prophylaxis of acute GVHD have over the years enabled better understanding of the side effects profile of mTOR inhibitors. Their potential to enhance generation of Tregs in vivo and thereby facilitate tolerance makes them promising agents in the first-line treatment of cGVHD [17, 18].

## Patients and methods

### Hypothesis and study objectives

The trial included patients from 6 transplant centres across Germany. Written informed consent was available from all patients. The study was approved by the Hamburg ethics committee and registered under ClinicalTrials.gov Identifier: NCT01862965.

The primary hypothesis of this study was that the addition of everolimus to prednisone increases response rates without increasing non-relapse and relapse mortality. The primary endpoint of the study was the rate of treatment success at 6 months (24 weeks) after initiation of treatment for cGVHD. Treatment success was defined as: the patient being alive at 6 months from study medication first intake and with no development of relapse of underlying disease and having achieved a complete remission (CR) or partial remission (PR) of cGVHD without addition of secondary systemic treatment for cGVHD. CR was defined as complete resolution of all symptoms attributed to cGVHD. PR was defined as one stage or more improvement in at least one organ without worsening in another. Secondary efficacy endpoints included time to first response and time to treatment failure.

Safety endpoints included assessment of the incidence of TMA, non-infectious pneumonitis (NIP), and avascular osteonecrosis.

Diagnosis and grading of cGvHD was according to NIH consensus-criteria [19]. Patient’s characteristics are summarized in Table 1. Patients received prednisone 1 mg/kg oral or intravenous for 2 weeks followed by dose reduction according to a recommended tapering schema (Supplementary Data). The initial dose of everolimus was 0.75 mg twice daily. The dose was adjusted to a targeted trough serum level of 3–8 µg/l, measured by HPLC or immunoassay four to 5 days after the previous dose change. If a patient was on a calcineurin inhibitor (CNI) at time of study inclusion, CNI was tapered and stopped within one to 4 weeks of initiation of study treatment, with an initial reduction of 50% at initiation of everolimus. The CNI baseline level was to be maximum 100 µg/ml for CSA and not higher than 6 µg/ml for tacrolimus after start of everolimus.

**Table 1:** Depicted are the baseline characteristics of all 34 patients in the efficacy analysis cohort. patient characteristics *n* = 34.

Age median (range)	55.5 (23–76)
Sex	
Male	21
Female	13
cGVHD severity at baseline	
moderate	21 (62%)
Severe	13 (38%)
cGVHD organ involvement	Total number (stage 1/2/3)
Eyes	12 (6/6/0)
Skin	32 (7/17/8)
Genitalia	4 (2/2/0)
GI tract	5 (4/1/0)
Joints and muscles	11 (6/4/1)
Liver	12 (8/1/3)
Lung	1 (1/0/0)
mouth	17 (10/6/1)
Skin phenotype	
Lichenoid / maculopapular	26
Sclerotic feature	6
Thrombocytopenia (<100/nl)	2
Overlap syndrome	2

Patients were treated for 1 year followed by a 1-year follow-up period. Patients discontinuing treatment prematurely or discontinuing treatment phase for reasons other than a recurrence of malignancies were censored at the date of study discontinuation.

### Inclusion and exclusion criteria

The study included adult patients 18 years or older with diagnosis of classic cGvHD according to NIH criteria and fulfillment of criteria for moderate or severe cGvHD, or diagnosis of overlap syndrome according to NIH criteria and fulfillment of criteria for moderate or severe cGvHD and ≤clinical grade 2 of acute GvHD of the gut and no grade 4 acute GvHD of the skin.

Patients were excluded from the trial as per SnPc if they had persistence, relapse or progression of underlying malignancy, uncontrolled infections or cytopenia with neutrophils <1000/µl and/or platelets <20,000/µl at time of screening. The full list of inclusion and exclusion criteria is included in Supplementary Data.

### Statistical analysis

Time to first treatment failure and speed of first response were analyzed using the Kaplan-Meier estimator. The number and percentage of patients who experienced death and who experienced relapse of underlying disease were calculated and a 95% binomial proportion CI was computed using the Wilson score method. Adverse events (AEs) were coded using the MedDRA® dictionary (version 15.1). Treatment failure and failure-free survival (FFS) were analyzed using the Kaplan-Meier estimator. Treatment failure was a composite event that included death, worsening or relapse of GvHD as well as relapse of underlying disease. The analysis was performed using the “survival” package of R software. The cumulative incidences of response were calculated using a competing risk method (Gray’s test) with death as a competing event. The analysis was performed with a “cmprsk” package of the R software.

## Results

**Fig. 1:**
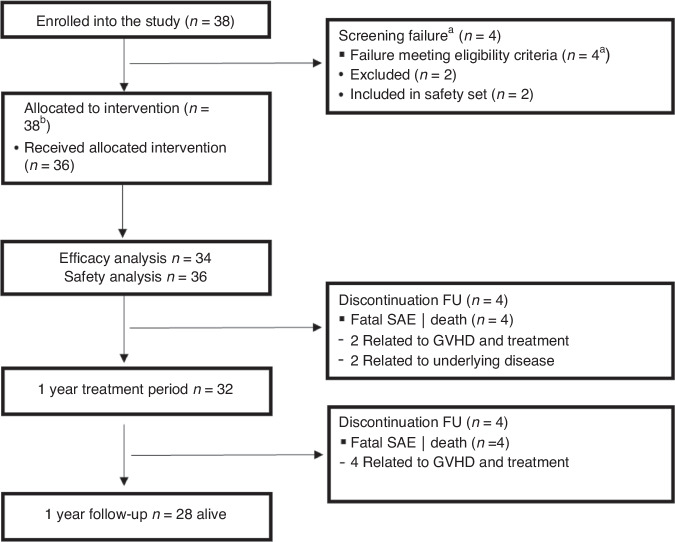
Consort diagram of study. FU follow-up.

A consort diagram of the study is shown in Fig. 1. The study included a total of 36 patients with moderate (*n* = 22, 61%) or severe (*n* = 14, 39%) cGVHD according to NIH. Median age was 55.5 (23–76) years, 13 patients were female and 23 male. Median time from diagnosis of cGVHD to study inclusion was 11 (range 1–126) days. Detailed organ involvement is depicted in Table 1. Two patients (in PR at time of exclusion) were excluded from the efficacy analysis because of screening failure (exclusion criteria pre-existing glaucoma and hypertriglyceridemia) but had received at least one dose of study treatment and thus included in the safety analysis set.

### Treatment efficacy

**Fig. 2:**
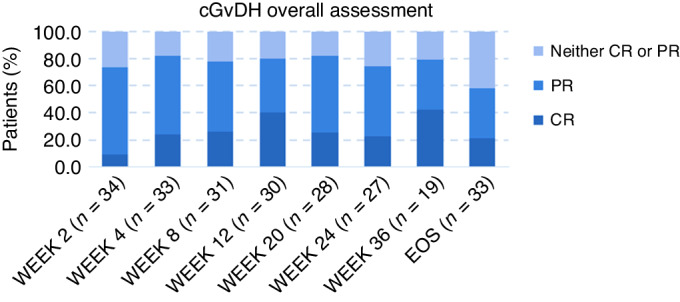
Depicted is the treatment response at various time points. CR complete remission, PR partial remission.

The efficacy analysis set included 34 patients with moderate (*n* = 21) or severe (*n* = 13) cGVHD. Overall, 30 of the 34 patients (88%) responded to study treatment with 14 (41%) CR and 16 (47%) PR. Proportions of patients responding at various time points are shown in Fig. 2.

**Fig. 3:**
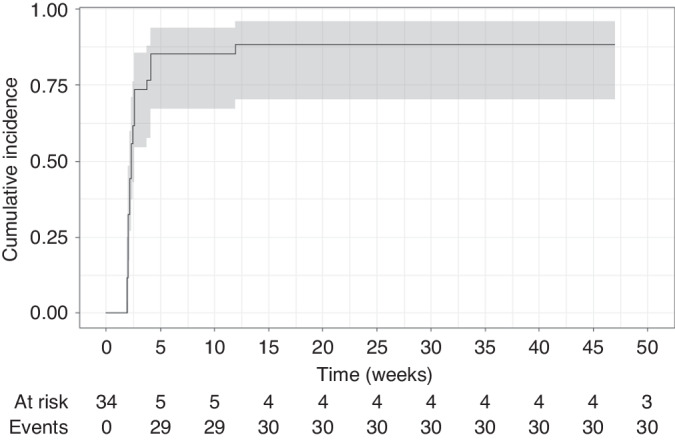
Cumulative incidence of treatment response. Depicted is the cumulative incidence of treatment response over time.

At 24 weeks (6 months) 19 out of 34 (56%, 95% CI: 39–71%) evaluable patients had achieved treatment success. Rate of treatment success was similar for patients with moderate (12/21 = 57%) or severe (7/13 = 54%) cGVHD. Nearly all responses (29/30) occurred within the first 6 weeks of treatment. Median time to first response was 2.3 weeks (Fig. 3). Median time to first response did not vary between patients with treatment success compared to those without treatment success (2.0 vs. 2.3 weeks). Rapid response was mainly driven by responses of cutaneous cGVHD with nearly 60% responding within the first 2 weeks compared to less than 20% for all other organs (Supplementary Fig. 1). At 2 weeks, only 2 out of 6 (33%) patients with sclerotic features compared to 17 out of 26 (65%) with only lichenoid/maculopapular lesions had responded to treatment.

**Fig. 4:**
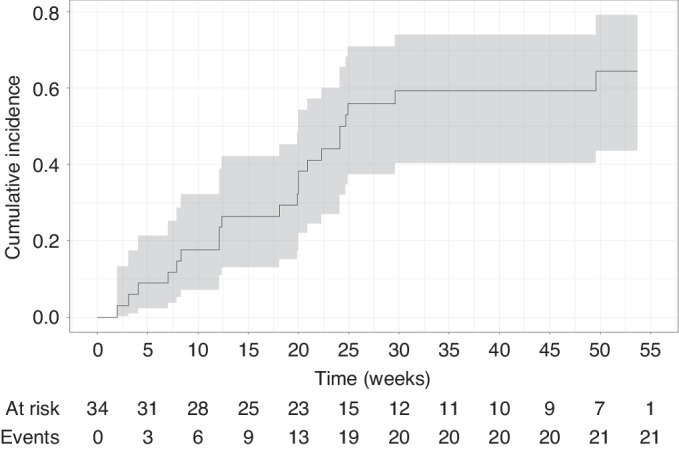
Cumulative incidence of treatment failure. Depicted is the cumulative incidence of treatment failure over time.

At 24 weeks, 15/34 (44.1%) patients had treatment failure. Reasons for treatment failure were need for secondary treatment due to lack of cGVHD response (*n* = 10) or due to side effects (*n* = 3), relapse of underlying malignancy (*n* = 1), death (*n* = 1). The median time to treatment failure was 24.7 weeks. A further 6 patients (17.6%) received secondary treatment between week 24 and week 52. At 52 weeks, 21/34 (62%, 95% CI: 46–80%) patients had treatment failure, corresponding to a 1-year treatment success and FFS rate of 38% (Fig. 4).

### Safety

All thirty-six (36) patients receiving at least one dose of the study treatment were included in the safety analysis set.

The most common AEs were infections (*n* = 27; 75%) hypertriglyceridemia (*n* = 17; 47%), followed by diarrhea (*n* = 14; 39%), and hyperglycemia (*n* = 10; 28%). The AEs of special interest as per secondary endpoints of the study including incidence of TMA, non-infections pneumonitis and avascular osteonecrosis were not observed in any patient.

During the course of the study, 45 AEs for 19 patients (53%) were assessed as serious (SAE) by the investigators. Most common SAEs were infections (*n* = 5; 14%). During the 1-year treatment period 4 patients died, 2 of the cases were considered related to study treatment, 1 from sepsis and the other from gastric hemorrhage. Two patients died due to relapse of underlying disease (myeloma in both cases). At the end of the 2-year treatment and follow-up period, 28 patients (77.8%) were alive while 8 (22.2%) patients had died due to relapse of underlying disease (*n* = 2) or complications of GVHD and its treatment (*n* = 6).

## Discussion

Clinically relevant endpoints are important for the evaluation of outcomes of patients treated for chronic GVHD. The primary endpoint, treatment success at 6 months was observed for 55.9% of the study patients with 6 more patients (17.6%) receiving secondary treatment between months 6 and month 12. For comparison, in the randomized phase II/III study evaluating efficacy of prednisone (PDN) and sirolimus (SRL) vs. PDN, SRL, and CNI in 138 patients with cGVHD [20] the primary endpoint was the proportion of patients being alive with CR or PR and without relapse or secondary therapy at month 6. In that study, which included about 20% patients with mild cGVHD, rate of treatment success was 48.6% (PDN/SRL) and 50.0% (PDN/SRL/CNI) at month 6. Like in our study, about 20–30% of patients receiving PDN/SRL and 11–24% receiving PDN/SRL/CNI received secondary treatment between month 6 and month 12. Recently, a prospective randomized trial reported a 6-months overall response and CR rates of 53 and 32% for patients treated with itacinib (400 mg qd) + steroids compared to 35 and 18% for steroids alone. The improvement in response rate, however, came at the expense of a higher risk of relapse of underlying malignancy and overall mortality [7].

In another study, Inamoto et al. used a novel composite endpoint, i.e., FFS which was defined as absence of second-line treatment, non-relapse mortality, and recurrent malignancy. This endpoint differs from the primary endpoint defined in our study, as it did not directly address treatment response (CR/PR) and furthermore, there was no direct predefined procedure to handle disease progression, in terms of second-line treatment initiation. The FFS rate was 68% after 6 months and 54% after 12 months, respectively [21].

Another study included a cohort of 328 patients that were enrolled within 3 months after diagnosis of cGVHD [22]. Patients received initial treatment for cGVHD including PRD with or without CNI (58%), PRD with or without CNI and other agents (29%), and other agents without PRD (13%). The study aimed to narrow down an endpoint that is associated with clinical benefit after initial treatment of cGVHD. They found that CR or PR at 1 year without secondary systemic treatment provides clinical benefit in patients with cGVHD. However, success as defined by that novel endpoint was reported to be currently observed for less than 20% of patients with cGVHD. Furthermore, conclusions made from results obtained at 6 months were found in that study to be less striking, especially as about 45–55% of the patients in that study received secondary systemic treatment between 6 months and 1 year [22].

Thus, the significance of the primary endpoint in this study might also be limited by the time point (month 6) for assessing treatment success. However, one of the secondary endpoints of this study was to access the time to treatment failure. At 1 year, treatment failure was observed in 63% of the patients indicating a treatment success rate of 37%, which appears to be higher than reported by Martin et al. (less than 20%).

Concerning the overall survival (OS) rate of patients treated with PDN and everolimus in our study, 78% of the patients were alive at 2 years. For comparison, Carpenter et al. observed OS rates at 2 years of 81.5% for PRD/SRL and 74% with PRD/SRL/CNI, respectively [20]. Similar rates were also observed by Martin et al., who reported survival rates of 87% of the patients in the control arm and 74% in the MMF arm, respectively [6].

Regarding relapse rate of underlying malignancies, 5.9% of the patients in our study experienced a relapse until study completion. These values are similar to results observed in other studies in which values of about 10–20% (81/400 and 32/328) of patients with recurrent diseases after 12 months were observed [7, 21, 22].

Regarding other safety aspects, incidence rates of the predefined safety endpoints TMA, NIP, and avascular osteonecrosis were low. In our study none of the patients experienced TMA. This is quite similar to low proportions observed in other studies that range from about 1% to about 5% [11, 20]. Likewise, no patients developed NIP. NIP is a known side effect of SRL and everolimus and values reported in literature show broad range of about 1% up to about 17% probably depending on target serum level and concomitant medication e.g., with CNI [20, 23–26].

Corticosteroids are considered a risk factor for the development of avascular osteonecrosis (AVN). None of the patients in this study showed AVN. Incidence rates reported in literature range from about 2 to 10% [27, 28]. The study published by McAvoy et al. investigated the corticosteroid dose dependent risk for avascular osteonecrosis risk; it revealed a 4.0 to 8.6 fold cumulative prednisone dose-dependent increased risk for patients receiving PDN [28]. Of note, in that study the median time from HCT to AVN was 15 (4–41) to 21 (1–80) months. Thus, significance of values observed in our study might be limited by the shorter time of observation.

Overall, despite the differences in the concrete definition of endpoints and the time point for assessing the (primary) endpoint(s) between the various studies, our data do not demonstrate beneficial effects from addition of everolimus to the first-line prednisone treatment regimen in terms of improvement of the primary endpoint (treatment success at 6 months). Addition of everolimus to prednisolone did not increase risk of relapse of underlying malignancy and was not associated with an increased risk of other side effects such as TMA and NIP or AVN. Notably the rate of treatment failure at 1 year was 63%, meaning 37% rate of treatment success, which appears higher than previously reported rates [22]. This is of particular importance because this endpoint is associated with clinical benefit [22]. Everolimus has a shorter terminal half-life of 28 h compared to 62 h for sirolimus and less toxic effects when combined with CNIs [29]. Furthermore, everolimus seems more effective in inhibiting class-I-stimulated mTORC2 [30], it is however unknown, whether this would have impact on treatment of GVHD. Larger randomized studies with even longer follow-up may help ascertain the role of everolimus in first-line treatment of cGVHD.

### Supplementary information

Supplementary data

## Data Availability

Data can be requested by e-mail to the corresponding author.
