# Supplementary material for: Treatment of newly diagnosed moderate or severe chronic graft-versus-host disease with prednisone and everolimus (PredEver first): a prospective multicenter phase IIA study
Source: Bone Marrow Transplant. 2024 May 2;59(8):1092–6. doi: 10.1038/s41409-024-02289-0 (PMC11296949; doi:10.1038/s41409-024-02289-0)

**Supplementary data**

### Inclusion criteria

Subjects eligible for inclusion in this study had to fulfill **all** of the following criteria:

1. Patient’s written informed consent
2. Women and men capable of reproduction must agree to use adequate contraceptive measures (condom, intrauterine devices, oral contraceptives) until three months after termination of treatment
3. Age ≥ 18 years
4. Diagnosis of classic cGvHD according to NIH criteria and fulfilment of criteria for moderate or severe cGvHD

or

Diagnosis of overlap syndrome according to NIH criteria and fulfilment of criteria for moderate or severe cGvHD and ≤ clinical grade 2 of acute GvHD of the gut and no grade 4 acute GvHD of the skin.

## Exclusion criteria

Subjects fulfilling **any** of the following criteria were not eligible for inclusion in this study:

1. Late persistent or recurrent acute GvHD without evidence of cGvHD
2. Relapsed or progressive malignant disease (other than minimal residual disease diagnosed by molecular methods)
3. Severe uncontrolled infections
4. Pregnant or lactating women
5. Inability to tolerate 1 mg/kg prednisone
6. Inability to take oral medication
7. Known hypersensitivity to everolimus
8. History of mTOR inhibitor associated non-infectious pneumonitis
9. Participation in another interventional clinical trial with intervention within < 30 days
10. Prior use of mTOR inhibitor (everolimus or sirolimus) for treatment of acute GvHD
11. Prior systemic treatment of cGvHD > 72 h. Patients treated for > 72h for cGVHD may be included in the trial if cGVHD was mild and no systemic steroids and/or mTOR-Inhibitors were used.
12. Psychiatric illness that would prevent granting of informed consent
13. Active viral infection with HIV, hepatitis B or hepatitis C
14. Severe cardiovascular disease (uncontrolled arrhythmias, congestive heart failure NYHA III or IV, or symptomatic ischemic heart disease)
15. History of mTOR inhibitor or CNI-associated TMA that led to discontinuation of mTOR inhibitor or CNI
16. Patients with neutrophils < 1,000/µl and/or platelets < 20,000/µl at time of screening
17. Donor lymphocyte infusion within the last 30 days
18. Pre-existing hyperlipidemia prior to treatment with calcineurin inhibitor or mTOR inhibitor
19. Wound healing complications
20. Active lymphoma as well as other malignancies
21. Edema (angioneurotic or peripheral)
22. Peptic ulcer
23. Severe colitis ulcerosa
24. Diverticulitis
25. Severe osteoporosis
26. Poorly controlled hypertension
27. Glaucoma (angle closure or open angle)
28. Cornea ulcer or cornea-injuries
29. Severe diabetes mellitus

Supplementary Table S1: Recommended tapering of prednisone in patients still responding or not responding after 2 weeks on 1 mg/kg body weight (BW)

| Week | Prednisone mg/kg BW | Comment |
| --- | --- | --- |
| 1-2 | 1 |  |
| 3-4 | 0.8 |  |
| 5-6 | 0.6 |  |
| 7-9 | 0.5 |  |
| 10-12 | 0.4 | *Week 12 evaluation for response. |
| 13-16 | 0.3 |  |
| 17-20 | 0.2 |  |
| 21-24 | 0.1 |  |
| 25-28 | 0.05 |  |
| 29-32 | 0.05 every other day |  |
| *In case of flare, tapering can be halted or prednisone increased two to three steps back.*  **non-responders are considered steroid refractory, responders continue on taper* | | |

Supplementary figure S1: Response over time according to organ


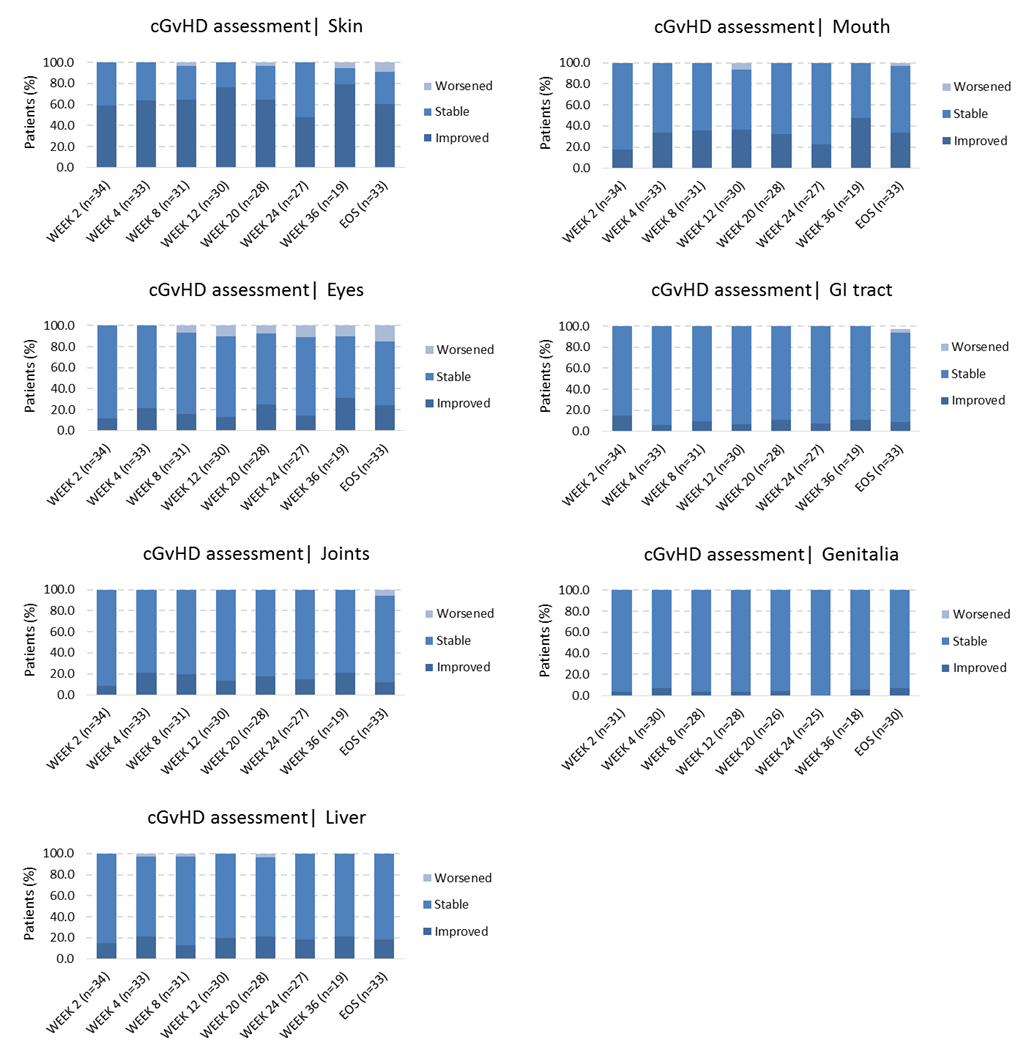


Supplementary figure S2: Failure free survival


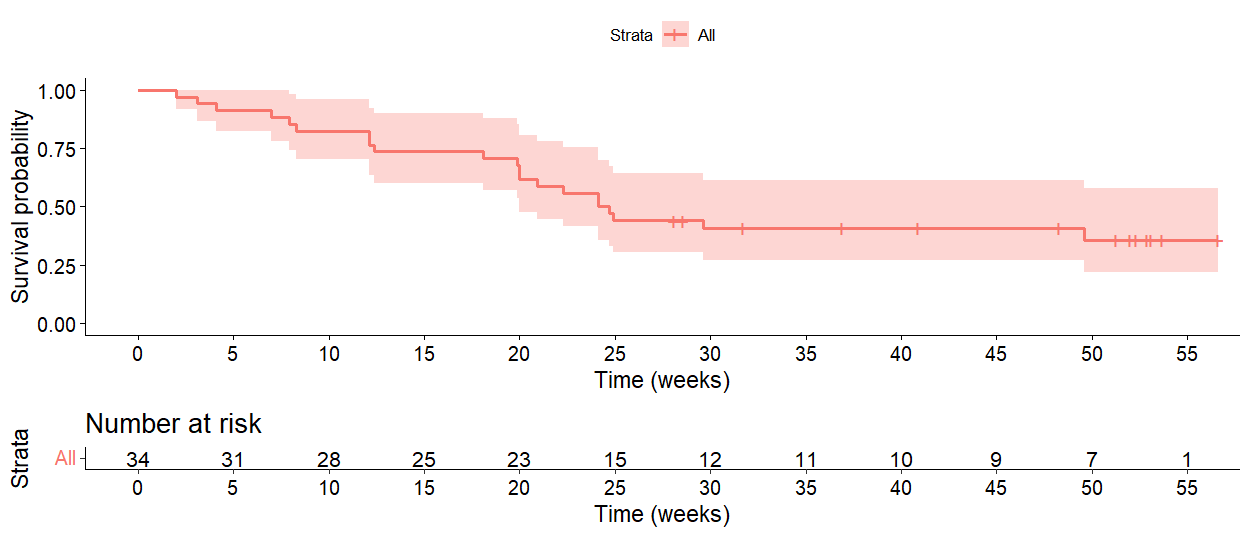

Supplement: Supplementary file 1 — Supplementary data [file 41409_2024_2289_MOESM1_ESM.docx]
